# Long Non-coding RNA LINC00847 Induced by E2F1 Accelerates Non-small Cell Lung Cancer Progression Through Targeting miR-147a/IFITM1 Axis

**DOI:** 10.3389/fmed.2021.663558

**Published:** 2021-04-22

**Authors:** Huan Li, Yao-kai Chen, Qiu Wan, An-qi Shi, Min Wang, Ping He, Li-xin Tang

**Affiliations:** ^1^Department of Infectious Diseases, Chongqing Public Health Medical Center, Chongqing, China; ^2^Department of Respiratory Geriatrics and Otolaryngology, Chongqing Public Health Medical Center, Chongqing, China; ^3^Department of Thoracic Surgery, Chongqing Southwest Hospital, Chongqing, China

**Keywords:** LncRNA LINC00847, NSCLC, IFITM1, E2F1, biomarker

## Abstract

**Background:** Long non-coding RNAs (lncRNAs) can remarkably regulate human malignancies in terms of the development and the progression. Previously, lncRNA LINC00847 (LINC00847) has been reported to present dysregulation in several tumors. However, the expression and function of LINC00847 in non-small cell lung cancer (NSCLC) have not been investigated.

**Methods:** RT-qPCR was performed to determine the expressions of LINC00847 in collected tissue samples and cell lines. The clinical significance of LINC00847 was statistically analyzed. CCK-8 test, cell scratch test and trans-well test were used to evaluate the proliferation, invasion and migration abilities of NSCLC cells, respectively. The xenograft tumor model was constructed to confirm the effects of LINC00847 knockdown on NSCLC *in vivo*. Further, luciferase reporter assays and Western blot were performed to explore molecular mechanisms underlying the functions of LINC00847.

**Results:** Increased expressions of LINC00847 were observed in NSCLC samples as well as cell lines. Additionally, E2F1 could be capable of directly binding to the LINC00847 promoter region, followed by promoting its expression. Clinically, LINC00847 high-expression could lead to poor prognosis of NSCLC patients. Functionally, LINC00847 knockdown noticeably repressed NSCLC cell growth and metastasis. Mechanically, miR-147a/IFITM1 axis was a downstream target of LINC00847, and silencing of miR-147a could rescue the anti-cancer effects of LINC00847 knockdown on NSCLC cell behaviors.

**Conclusion:** Overall, up regulation of LINC00847 induced by E2F1 promoted the progression of NSCLC by modulating miR-147a/IFITM1 axis, representing a novel regulatory mechanism for NSCLC progression.

## Introduction

Lung cancer can be seen as the major cause of cancer-related death globally, thereinto, non-small cell lung cancer (NSCLC) occupies about 85% (1–3). Over one-third of new NSCLC cases could be found in China, which was a heavy burden facing patients, their families, the society and the whole country (4). Despite the big progress in terms of the diagnosis and the therapy of NSCLC recently, it faces a poor 5-year overall survival (OS) because most patients are diagnosed at advanced stages with lymphatic metastasis, thereby limiting the successful treatment (5, 6). Therefore, identifying novel NSCLC biomarkers as well as better figuring out the potential molecular mechanisms of NSCLC can assist in improving NSCLC diagnosis and treatment.

Long non-coding RNAs (lncRNAs) refer to a cluster of evolutionarily conserved ncRNAs which are over 200 nucleotides long, with limited or without protein-coding capacity (7). The lncRNAs which have been found newly show tissue-specific expression patterns. Based on new evidence, lncRNAs have different functions in different biological activities, particularly in the development process of tumors (8, 9). It has been demonstrated that lncRNAs act as potential regulators in the expression of tumor-related genes, which suggested, together with their frequent dysregulation, that lncRNAs could effectively affect the diagnosis of tumors as well as be an effective therapeutic target (10, 11). For instance, lncRNA HOXA11-AS was highly expressed in lung cancer, and its overexpression facilitated the proliferation and metastasis of lung cancer cells via miR-148a-3p/DNMT1 axis (12). LncRNA LOXL1-AS1 knockdown hindered the ability of cell growth and invasion through increasing MYBL2 expression via sponging miRNA-423-5p (13). However, the expression and function of many lncRNAs have not been investigated.

LncRNA LINC00847 (LINC00847), located on 5q35.3, was a newly identified lncRNA. In recent years, the abnormal expressions of LINC00847 have been reported in several tumors, such as renal cell carcinoma, breast carcinoma and lung cancer (14–16). However, those results were based on the level of a small size of samples, and the functional assays of LINC00847 in tumors have not been performed. The study contributed to clinical evidences to prove LINC00847 as a kind of overexpressed lncRNA in NSCLC. Also, many *in vitro* assays were conducted to explore its tumor-related function.

## Materials and Methods

### Patients and Specimens

We obtained paired NSCLC specimens together with adjacent non-neoplasm lung specimens from 70 patients receiving curative surgical removal in Chongqing Public Health Medical Center between July 2015 and December 2017. All patients provided written informed consent. The clinical diagnosis for all NSCLC patients was confirmed by two pathologists who make their own judgment independently. The clinic pathological data of all 70 patients were reviewed and collected from The Follow-up Department, and TNM stages of tumors were classified based on the eighth edition AJCC system. The resent study has obtained the approval of the Ethics Committee of Chongqing Public Health Medical.

### Cell Culture, Cell Transfection and RNA Interference

We obtained human NSCLC cell lines (SK-MES-1, Calu-3, A549 and H460 cells) from the American Type Culture Collection (ATCC, Rockville, MD, USA). The normal human bronchial epithelial (NHBE) cells were provided by the Shanghai Institute of Cell Biology. All cells were grown in RPMI-1,640 medium, which contained 10% fetal bovine serum (FBS, Biocyto Technology, Daxuecheng, Guangzhou, China), 1.2% antibiotics (Boaosen, Haidian, Beijing, Chian) and 1% glutamate (Biocyto Technology) followed by incubation at 37°C.

SiRNAs targeting E2F1 (si-E2F1), LINC00847(si-LINC00847-1, si-LINC00847-#2), miR-147a mimics, miR-147a inhibitors came from GEkai Teconology (Xuhui, Shanghai, China). Experimenters cloned LINC00847 or E2F1 into pcDNA3.1 empty vector for the consistent expression of LINC00847 or E2F1, respectively. Lipofectamine 3,000 reagents (Fujun, Haidian, Beijing, China) assisted in transfecting cells following the kits' protocols.

### RNA Isolation and RT-PCR Assays

Total RNA was extracted from specimen samples as well as above cells by TRIzol (Kehaojia, Hongshan, Wuhan, China) containing 0.2 ml chloroform (Hengdu, Pudong, Shanghai, China). Reverse transcription of RNA was performed to obtain cDNA templates by the use of Super M-MLV (Baitaike, PR6502, Pudong, Shanghai, China). For the determination of lncRNA, miRNA and mRNAs, qPCR was conducted using the SYBR Green PCR Kit on a BioRad Chromo4 PCR system (Biosystems, Foster City, CA, USA). GADPH or U6 served as an endogenous control. 2^−ΔΔ*Ct*^ method was applied to calculate the levels of the above factors. The primer was synthetized by Kairuiji Technology (Shijingshan, Beijing, China), and its sequences were presented in Table 1.

**Table 1:** The primers used in this study for RT-PCR.

**Genes**	**Sequences (5′−3′)**
LINC00847: forward	TGACCATCAGAATGGCAGATA
LINC00847: reverse	GACCCTGACGCTGTCGATCAA
E2F1: forward	AGCGGCGCATCTATGACATC
E2F1: reverse	GTCAACCCCTCAAGCCGTC
miR-147a: forward	CCCCTATCACGATTAGCATTAA
miR-147a: reverse	CCCAAGCTTTTATGTGGTTGTT
IFITM1: forward	TCGCCTACTCCGTGAAGTCTA
IFITM1: reverse	TGTCACAGAGCCGAATACCAG
GAPDH: forward	TATAAATTGAGCCCGCAGCC
GAPDH: reverse	TACGACCAAATCCGTTGACTC
U6: forward	GCGCGTCGTGAAGCGTTC
U6: reverse	GTGCAGGGTCCGAGGT

### Luciferase Reporter Assay

JASPAR was used to identify the E2F1-binding motif in LINC00847 promoter region. The divergent fragment sequences were synthesized by Jima Technology (Hangzhou, Zhejiang, China), followed by being inserted into pGL3-basic vector (Promega, Haidian, Beijing, China) and co-transfected with E2F1 plasmid into Calu-3 and A549 cells. The miR-147a sequence was also inserted into pGL3-basic vector and wild-type and mutant LINC00847 plasmid were used for co-transfection. Luciferase activities were measured 24 h later using the dual luciferase reporter system (Promega, Madison, WI, USA). Renilla luciferase activity was used as a standardized control.

### RNA Pull-Down Assays

A Magnetic RNA Protein Pull-Down Kit (Pierce, USA) was applied to the RNA pull-down assay following the manual.

### Subcellular Fractionation

The PARIS Kit (Life Technologies, USA) was applied to the subcellular fractionation (separating nuclear from cytoplasmic) following the instruction of the producer.

### Cell Proliferation Assays

CCK-8 assays as well as colony formation assays were carried out for determining the proliferative ability of tumor cells after the special treatments in different conditions. For CCK-8 assays, we seeded cells in 48-well plates (1,500 cells per well) which were maintained at 37°C for 4 days. CCK-8 reagent was added at the setting time after cell seeding. The viable cells were assessed by measuring the absorbance at 450 nm. For the colony formation assays, tumor cells (300 cells per well in a 24-well plate) were placed in a 6-well plate and cultured with RPMI 1,640 medium (GIBCO, Haidian, Beijing, China) for 14 days. The colony formation was visualized by crystal violet staining.

### Colony Formation Assays

We seeded 3,000 cells on each well of a 6-well plate, and used individual siRNA oligonucleotides to transfect them. The medium was changed each 4 days. Two weeks later, methanol was employed to fix the colonies, which then received 15 min of staining treatment in 0.1 % crystal violet (Sigma, Pudong, Shanghai, China) in PBS. We captured the images of these colonies containing ≥50 cells and used ImageJ (V.1.8.0) software for counting.

### Ethynyldeoxyuridine (EdU) Assays

Cell-Light EdU Apollo 567 *in vitro* Imaging Kit (Ribobio Technology, Guangzhou, China) served for the EdU assays following the instruction of the producer. Fluorescent microscopy (Nikon, Japan) assisted in measuring the percentage occupied by EdU-positive cells in the five random fields each well. The experiments were conducted repeatedly in triplicate.

### *In vivo* Assay

To explore the potential function of LINC00847 on tumor growth, 5–6 week old female athymic nude mice (BALB/c Nude) were used for the xenograft model (*n* = 6 per group). A549 cells stably transfected with sh-control and sh-LINC00847-1 were dissociated using trypsin and washed twice with sterilized PBS. Then, 0.3 mL of PBS containing 3 × 10^6^ cells was subcutaneously inoculated into the flank of mice. The tumor size was determined every 3–4 days after tumor formed (around 1–2 weeks). The tumor volumes were recorded every 4 days and calculated in accordance with a formula (length × width^2^ × 0.5). The animal-related protocol was approved by the Animal Research Ethics Committee of Chongqing Public Health Medical Center.

### Cell Wound Healing

Each well of a 6-well plate was added with approximately 4 × 10^5^ cells, which were cultured until completely confluent. Then, in a gentle way, a 20-μl micropipette tip was used to scratch cells in the center of the well. At 0 and 48 h incubation, the NSCLC cell lines were photographed using the inverted microscope (Olympus, Japan) and the scratch area was assessed using Image J software.

### Trans Well Assay

The trans well assay was conducted for the functional assays of LINC00847 using the 24-well trans wells precoated with the Matrigel (Solarbio, Hengfei Technology, Shanghai, China). Serum-free RPMI-1,640 assisted in seeding Calu-3 and A549 cells (5,000) into the upper chamber. The lower chamber was added with medium (which contained 10% FBS) as the chemical attractant. Then, a cotton swab was used to remove the cells that remained on membrane upper surface. Following 24 h of incubation, methanol was used to fix invaded cells. Then 1% crystal violet solution was used to stain the invaded cells. At last, a light microscope served for calculating invaded cells in five random view fields.

### Western Blot Assays

Total protein from NSCLC cells was obtained by applying RIPA lysis buffer (Sigma, Hangzhou, Zhejiang China) with protease suppressor cocktail (Roche, Pudong, Shanghai, China). Polyacrylamide gel electrophoresis (10%) assisted in resolving 30μg protein, followed by PVDF membrane transferring (Millipore, Chida Technology, Xuhui, Shanghai, China) in ice bath. Next, the membranes were blocked for 2 h by 5% BSA diluted with TBST at 37 °C, which were further probed by using the primary antibodies for one night at 4 °C. Subsequently, PBST which contained anti-rabbit-horseradish peroxidase-conjugated secondary antibodies (Sangong, Hangzhou, Zhejiang, China) was used to incubate the membranes for 60 min at 37 °C. Densitometry was performed using ImageJ software. The primary antibodies were purchased from Haodi Technology (Shenzhen, Guangdong, China).

### Statistical Analysis

SPSS 21.0 (SPSS Inc., Chicago, IL, USA) together with GraphPad Prism 8.0 software were applied for statistical calculation and graphing, respectively. Student's *t*-test was applied to compare the two groups. The chi-square test assisted in analyzing the clinical value possessed by LINC00847 in NSCLC patients. The Kaplan-Meier method was applied for analyzing the survival, and the Log-rank (Mantel-Cox) test served for determining the between-group differences in survivals. Univariate and multivariate analyses for the examination of the prognostic value of LINC00847 were based on the Cox regression model. A *p* < 0.05 exhibited statistical significance.

## Results

### LINC00847 Expression Level Was Overexpressed in NSCLC Samples

**Figure 1:**
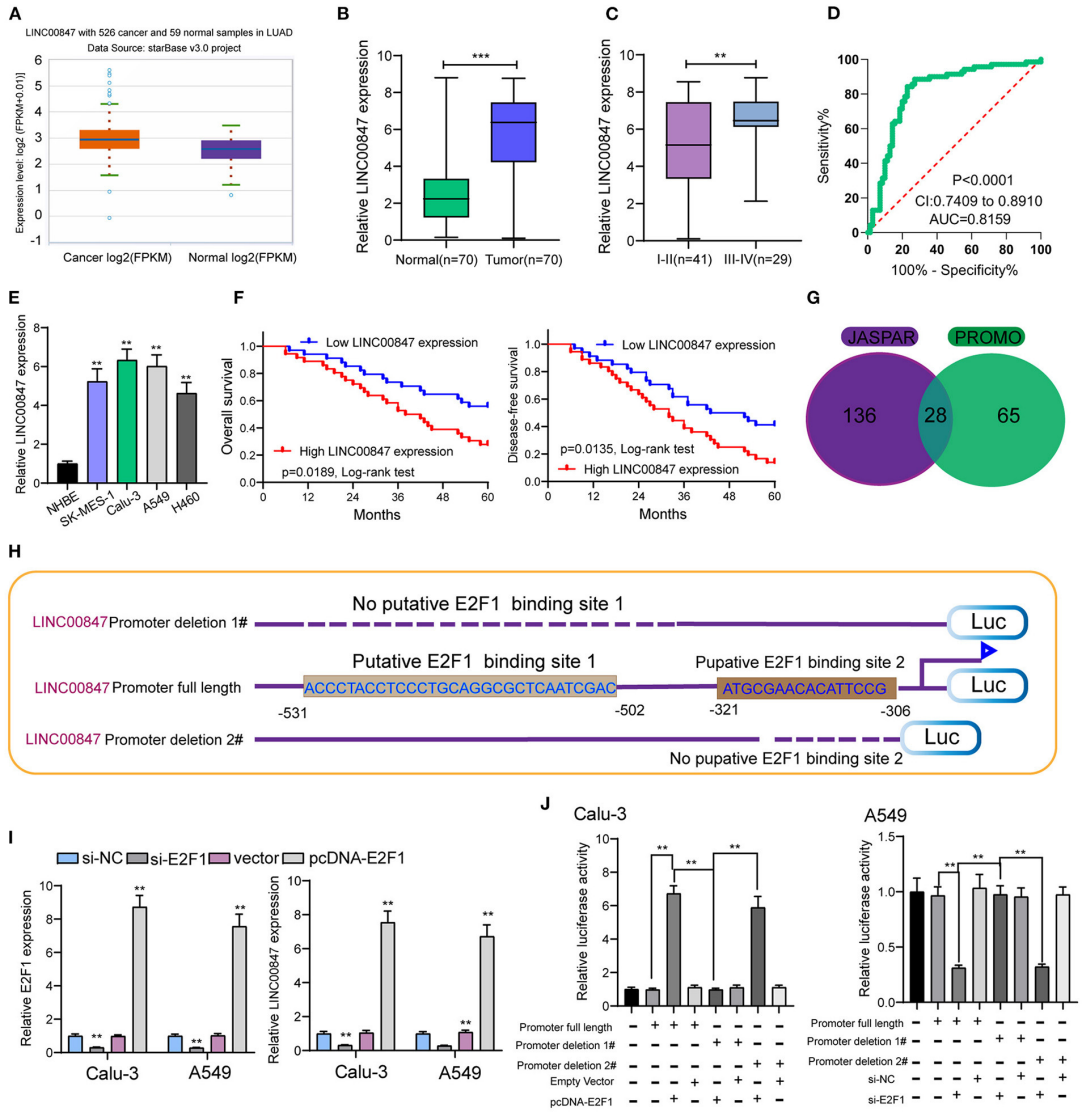
LINC00847 expression was up regulated in NSCLC and induced by E2F1. **(A)** The expressing trend of LINC00847 in lung cancer and normal lung samples of TCGA datasets. **(B)** Relative expressions of LINC00847 in 70 NSCLC tissue samples and their paired non-cancerous specimen samples measured by RT-qPCR. **(C)** LINC00847 expression was higher in advanced NSCLC specimens. **(D)** ROC assays for the exploration of the diagnostic value of LINC00847 expression in NSCLC specimens. **(E)** RT-PCR for the determination of LINC00847 levels in four NSCLC cell lines and NHBE. **(F)** Kaplan-Meier curves for the overall survival and disease-free survival in patients with NSCLC. **(G)** 28 transcription factors were predicted for LINC00847 using JASPAR and PROMO databases. **(H)** Description of E2F1-binding sites in the promoter region of LINC00847 and the schematic of LINC00847 promoter deletion 1# and LINC00847 promoter deletion 2# luciferase reporter vectors. **(I)** The expression of LINC00847 after knockdown or overexpression of E2F1. **(J)** Relative luciferase activities as analyzed in Calu-3 and A549 cells co-transfected with the related vectors. **p < 0.01.

For the exploration of the function of LINC00847 expression in NSCLC progression, we searched starbase 3.0(http://starbase.sysu.edu.cn/) which is an online tool and can be used to analyze TCGA datasets. As shown in Figure 1A, we observed that LINC00847 is an overexpressed lncRNA in NSCLC (Figure 1A). As demonstrated by RT-PCR results, higher LINC00847 levels were observed in NSCLC specimens than matched normal specimens (*p* < 0.01, Figure 1B). Also, the NSCLC specimens with advanced stages exhibited a higher level of LINC00847 (Figure 1C). According to the ROC assays, our group observed that high LINC00847 expression had an AUC value of 0.7951(95% CI: 0.7447–0.8456) for NSCLC (Figure 1D). Next, our group tested LINC00847 expression in NSCLC cell lines, finding that LINC00847 was more expressed in NSCLC cell lines (Figure 1E).

### High-Expressions of LINC00847 Predicts Unfavorable Prognosis in NSCLC Patients

Given the fact that the distinct up regulation of LINC00847 was observed in NSCLC tissues, we wondered whether its levels may display an influence on the clinical progress of NSCLC patients. LINC00847 expression value was taken as a standard to divide enrolled patients into two groups, and chi-square test which indicated the up regulation of LINC00847 expressions showed an obvious relation to clinical stage (*p* = 0.047) and lymph nodes metastasis (*p* = 0.041, Table 2). Moreover, we further performed Kaplan-Meier analysis for confirming the prognostic value owned by LINC00847 in NSCLC patients. It was observed that the patients with high LINC00847 expression displayed evidently poorer OS (*p* = 0.0189) and disease-free survival (DFS) (*p* < 0.0135) relative to lower LINC00847 expressions (Figure 1F). Finally, as revealed by multivariate Cox regression analyses, LINC00847 expression may independently predict the OS and DFS of NSCLC patients (Table 3).

**Table 2:** Correlation between LINC00847 expression and clinicopathological characteristics of NSCLC patients.

**Variable**	**Number**	**LINC00847 expression**		***p***
		**High**	**Low**	
Age (years)				0.229
< 55	36	16	20	
≥ 55	34	20	14	
Gender				0.663
Male	43	23	20	
Female	27	13	14	
Tumor size (cm)				0.134
< 3	41	18	23	
≥ 3	29	18	11	
Histology				0.650
Adeno	39	21	18	
Squamous	31	15	16	
Smoking history				0.967
Smokers	41	21	20	
Never smoke	29	15	14	
Clinical stages				0.047
I-II	41	17	24	
III-IV	29	19	10	
TNM stage				—
I(T1-2N0M0)	36	15	21	
II(T1-2N1M0)	5	2	3	
IIIA (T3N0M0;T1-3N2M0)	17	8	9	
IIIB(T1-4N3M0;T4N1M0)	9	8	1	
IV(T1-4N1-3M1)	3	3	0	
Lymph nodes metastasis				0.041
No	48	21	27	
Yes	22	15	7	

**Table 3:** Summary of multivariate Cox regression analyses of overall survival and disease-free survival duration.

**Parameter**	**Overall survival**			**Disease-free survival**		
	**HR**	**95% CI**	***p***	**HR**	**95% CI**	***p***
Age	0.893	0.463–1.773	0.232	1.241	0.664–2.311	0.188
Gender	1.215	0.667–2.152	0.188	1.452	0.783–1.987	0.267
Tumor size	1.427	0.782–2.441	0.139	1.118	0.673–2.231	0.109
Histology	1.576	0.875–2.774	0.119	1.334	0.678–2.213	0.342
Smoking history	1.334	0.673–1.893	0.231	0.986	0.674–1.783	0.134
Clinical stage	2.986	1.376–4.782	0.011	2.675	1.175–4.376	0.019
Lymph nodes metastasis	3.328	1.477–5.271	0.006	2.986	1.218–4.778	0.009
LINC00847 expression	2.896	1.476–5.221	0.009	2.562	1.217–4.562	0.018

### E2F1 Activates LINC00847 Transcription in NSCLC Cells

For figuring out the mechanism involved in LINC00847 overexpression in NSCLC cells, we analyzed the JASPAR tool for the determination of possible transcription factors binding to the LINC00847 promoter. As shown in Figure 1G, we found several transcription factors which may be the potential candidates, such as YY1, ZNF354C, ZNF263 and E2F1. We focused on E2F1 because several E2F1 binding sites displayed high scores and E2F1 have been demonstrated to be highly expressed in lung cancer and served as a tumor pro motor(Figure 1H) (17, 18). Then, the expression of LINC00847 was silenced or overexpressed and RT-PCR was performed, which suggested that the transfection of si-E2F1 could suppress the levels of both E2F1 and LINC00847 in A549 and calu-3 cells; by contrast, E2F1 overexpression showed a positive effect (Figure 1I). Dual luciferase reporter assays revealed that the promoter activity reduced remarkably due to LINC00847 promoter deletion 1# relative to the full-length promoter construct and LINC00847 promoter deletion 2# (Figure 1J). Our data revealed that E2F1 transcriptionally activated LINC00847 and promoted its levels in NSCLC cells.

### Knockdown of LINC00847 in NSCLC Decreased Cell Growth

**Figure 2:**
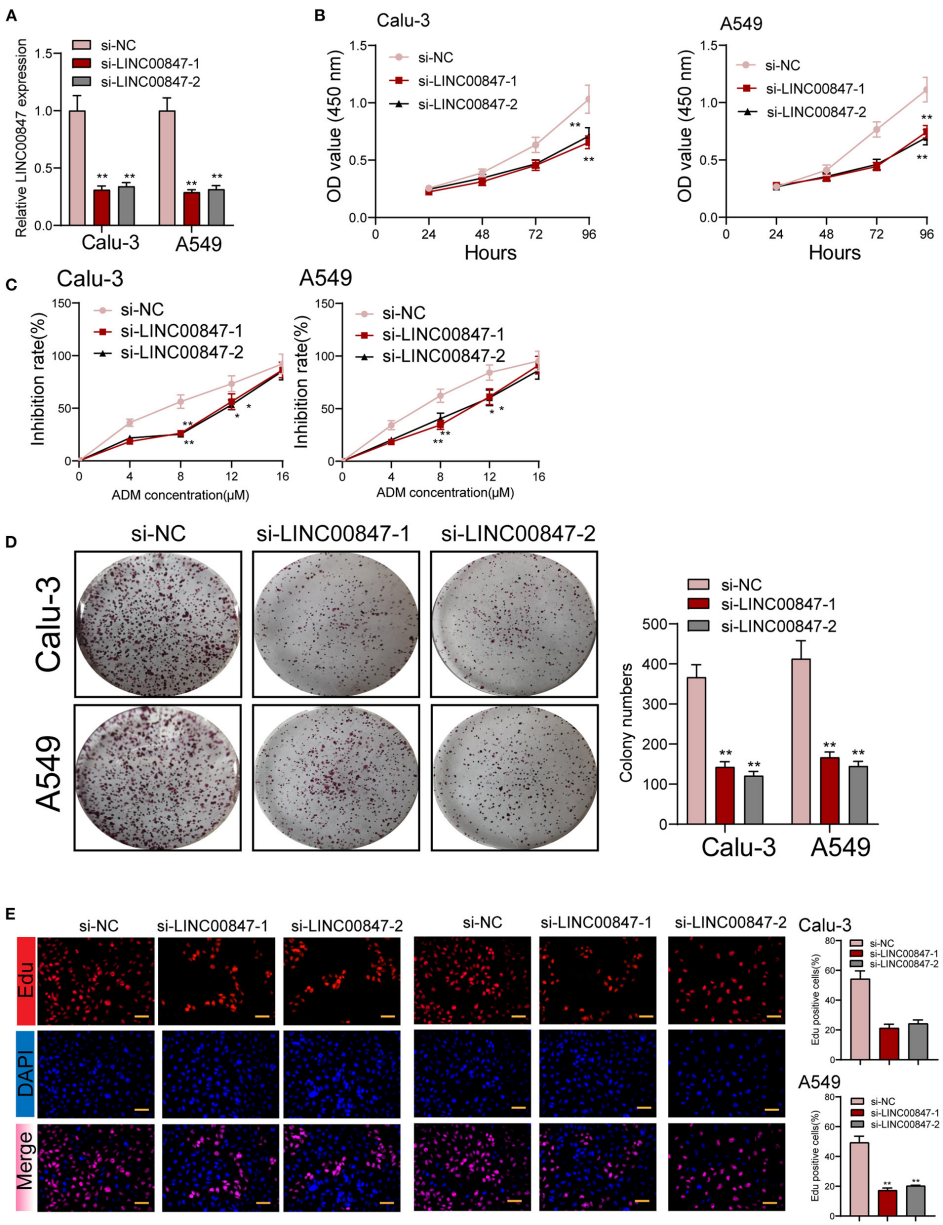
The proliferation of NSCLC is promoted by LINC00847. **(A)** Relative expression of LINC00847 in NSCLC cells transfected with LINC00847 siRNA (si-LINC00847-1 or si-LINC00847-2) and scrambled siRNA. **(B)** CCK8 assay was used to detect cell viability. **(C)** Calu-3 and A549 cells were treated with ADM (0, 4,8,12,16μM) for 48 h. Inhibition rate increased with the increase of concentration of ADM. **(D)** LINC00847 knockdown remarkably impaired the ability of colony formation. **(E)** EDU assays for the determination of the cell proliferation changes after LINC00847 down-regulation in Calu-3 and A549 cells. *p < 0.05, **p < 0.01.

**Figure 3:**
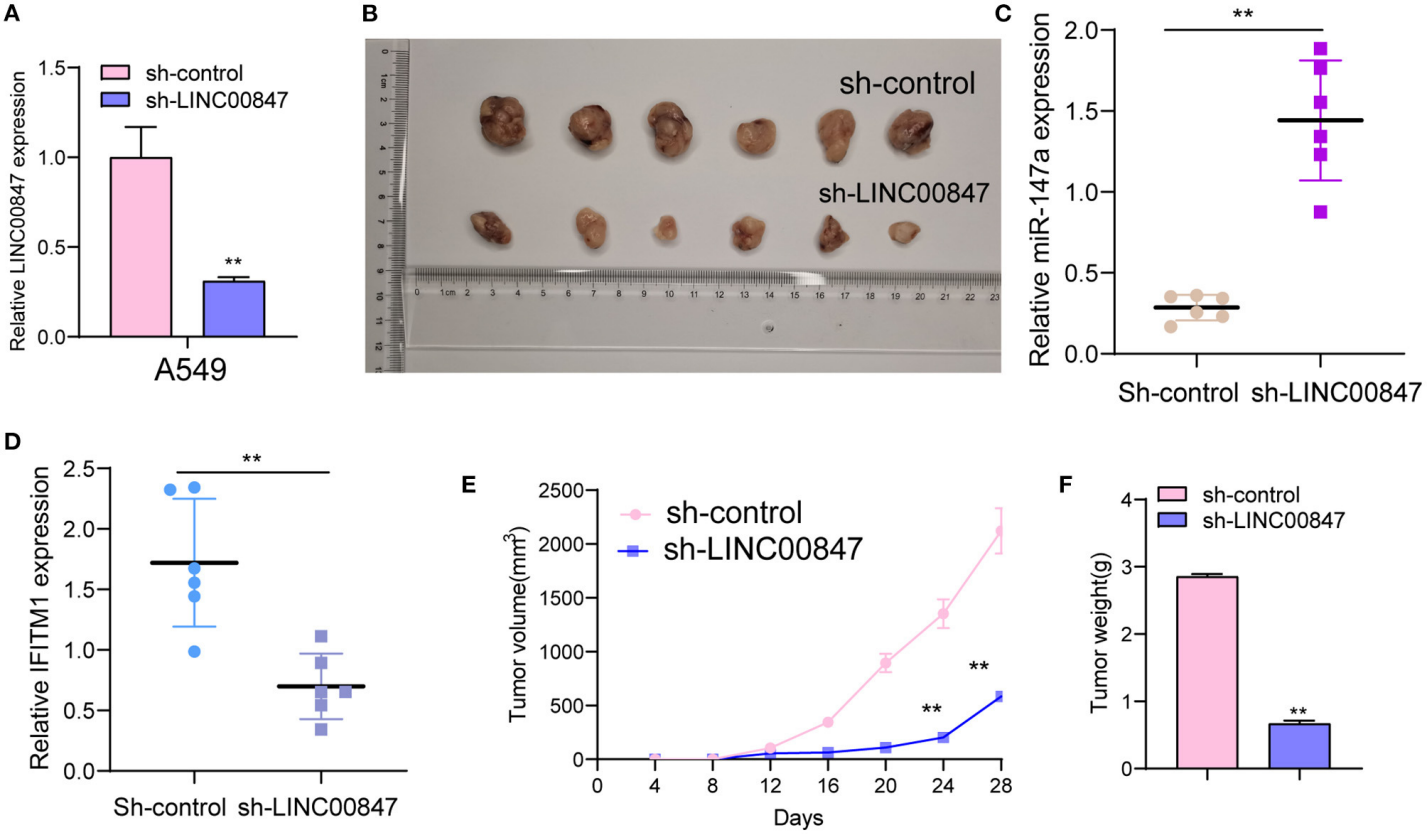
LINC00847 promoted tumor growth *in vivo*. **(A)** The distinct knockdown of LINC00847 expression in A549 cells transfected with LINC00847 were demonstrated by RT-PCR. **(B)** Tumors derived from mice in two different groups were presented. **(C, D)** RT-PCR determined the expression of miR-147a and IFITM1 in tumors with sh-control and sh-LINC00847. **(E)** Tumor growth curves were shown every 7 days. **(F)** The subcutaneous tumor weights were detected at the 28th day after injection. **p < 0.01.

The frequent overexpression of LINC00847 and its effects on enhancing tumor metastasis in clinical progress encouraged us to further explore the possible biological function in the NSCLC behaviors. Using siRNA, LINC00847 expression was silenced in Calu-3 and A549 cells (Figure 2A). CCK8 assays suggested that cell growth was distinctly inhibited in both cell lines transfected with si-LINC00847 compared with si-NC (Figure 2B). The study also paid attention to exploring how LINC00847 affected the chemotherapy resistance exhibited by NSCLC cells, finding that ADM concentration increase led to increased inhibition rate, and LINC00847 knockdown weakened the inhibition effect imposed by ADM specific to NSCLC cells (Figure 2C). In addition, we also observed that colony-formation ability was decreased by LINC00847 knockdown in NSCLC cells (Figure 2D). In line with EDU staining, there were fewer EDU-positive cells in si-LINC00847-1 and si-LINC00847-2 group compared with the si-NC group (Figure 2E). Then, *in vivo* experiments were used to further explore the potential influence of LINC00847 inhibition on tumor growth. Figure 3A confirmed the transfection efficiency of sh-LINC00847. *In vivo* assays suggested that depletion of LINC00847 distinctly decreased tumor growth rate (Figure 3B). We also examine the levels of miR-147a and IFITM1 in these tumors, finding that miR-147a expression in tumors with sh-LINC00847 group was distinct higher than those with tumors with sh-NC group, while a significant decrease of IFITM1 expression was observed in tumors with sh-LINC00847 group compared with those with sh-NC group (Figures 3C,D). In addition, our group showed that the tumor volume and weight were apparently lessened in sh-LINC00847 group compared with control group (Figures 3E,F). Overall, our findings suggested that knockdown of LINC00847 suppressed NSCLC tumorigenesis *in vivo*.

### Silencing LINC00847 Inhibits Invasion and Migration of NSCLC Cells

**Figure 4:**
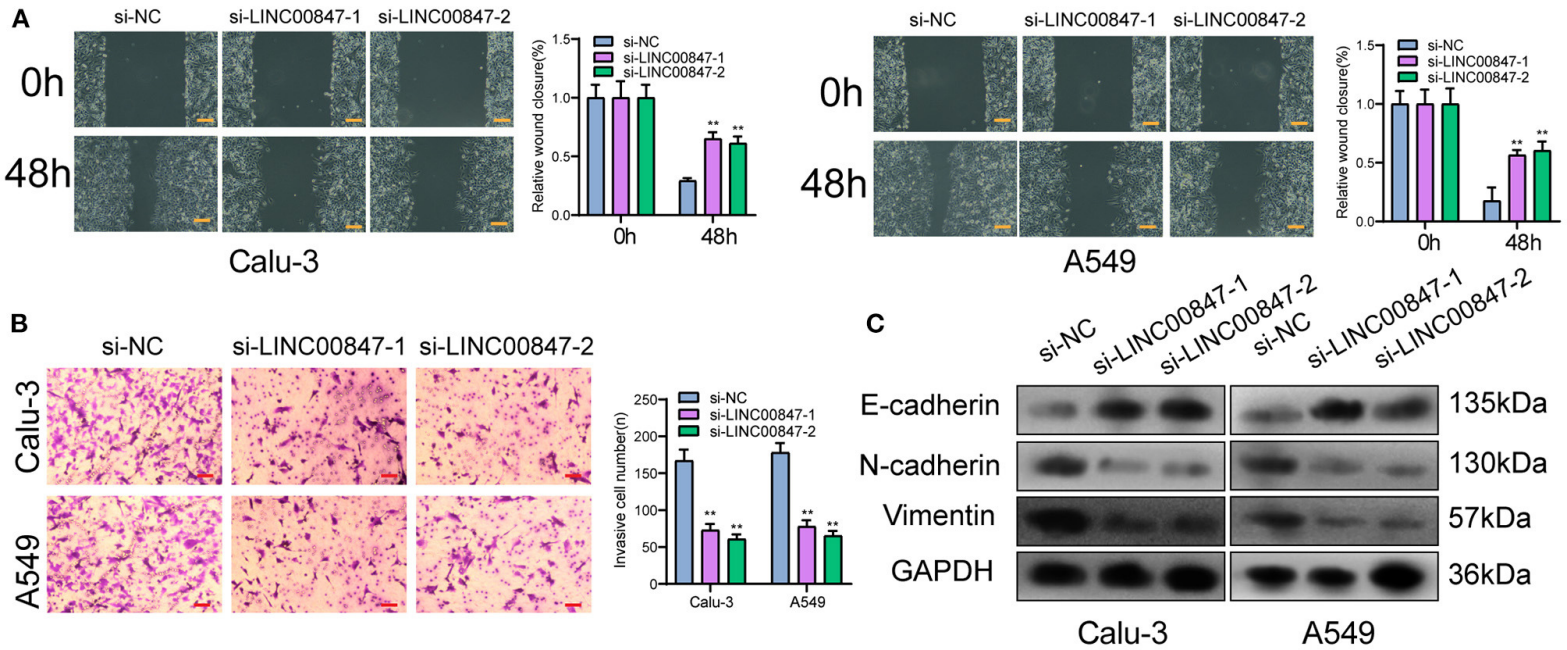
LINC00847 dramatically influenced the invasion, migration and EMT progress of NSCLC cells. **(A)** Wound healing assays were used to detect cell invasion in LINC00847-knockdown-Calu-3 and A549 cells. **(B)** Trans well assays were carried out in Calu-3 and A549 cells after knockdown of LINC00847. **(C)** Western blot examined the levels of EMT-related factors after knockdown of LINC00847. **p < 0.01.

The wound-healing and trans well assays served for further examining the ability of cell metastasis. In line with Figure 4A, the migratory areas of si-LINC00847 in Calu-3 and A549 cells were distinctly smaller than those of si-NC. In addition, the results of trans well assays indicated that the si-LINC00847 group had a significantly decreased cell invasion ability relative to the si-NC group (Figure 4B). Besides, to explore the metastatic mechanism involved in LINC00847 function, we performed Western blot for detecting the activity exhibited by epithelial-mesenchymal transition (EMT)-related markers. As shown in Figure 4C, the suppression of LINC00847 distinctly reduced the N-cadherin and Vimentin levels as well as improved the E-cadherin levels in both Calu-3 and A549 cells. Our data revealed that LINC00847 may promote metastasis and EMT progress in NSCLC cells.

### LINC00847 Directly Interacts With miR-147a and Represses Its Expression

**Figure 5:**
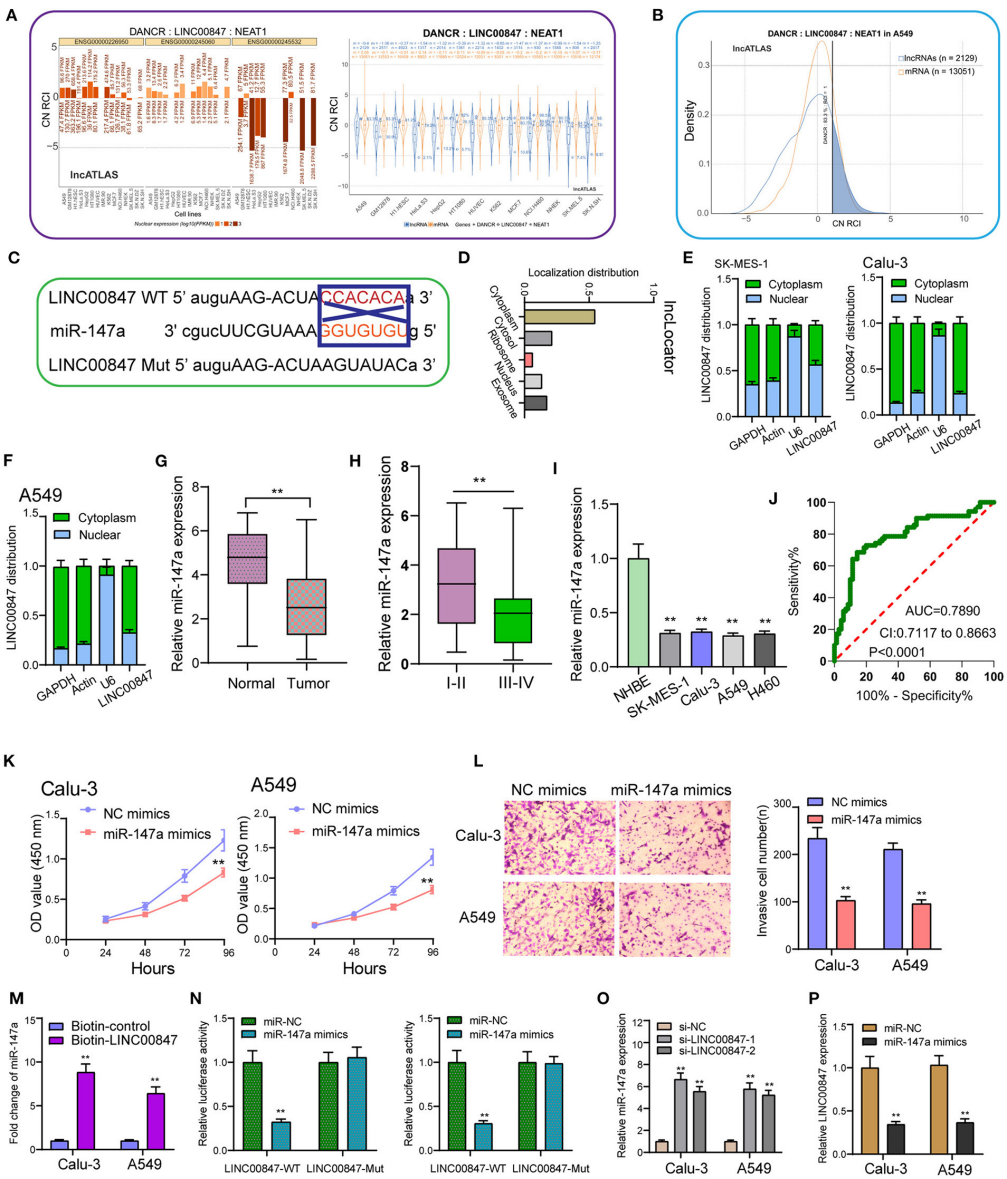
miR-147a is predicted to interact with LINC00847. **(A)** The subcellular localization of LINC00847 in several types of tumor cells based on the data of LncATLAS. **(B)** Cytoplasmic/Nuclear Localization: RCI distribution in A549 cells. **(C)** The schematic diagram presents the complementary binding sites within LINC00847 and miR-147a. **(D)** Localization distribution of LINC00847 by analyzing lncLoctor. **(E, F)** Relative LINC00847 expression levels in nuclear and cytosolic fractions of SK-MES-1, Calu-3 and A549 cells. **(G)** RT-PCR for the determination of miR-147a expression in our cohort. **(H)** The levels of miR-147a in NSCLC with different stages. **(I)** The down-regulation of miR-147a was observed in NSCLC cells. **(J)** The diagnostic value of miR-147a was determined using ROC assays. **(K)** CCK-8 assays determined the proliferation ability of Calu-3 and A549 cells after transfection of miR-147a mimics. **(L)** Trans well-assay was used to detect cell invasion in miR-147a mimics transfected Calu-3 and A549 cells. **(M)** RNA-pull down experiments suggested the combination between miR-147a and LINC00847. **(N)** Luciferase reporter assay showed the activity within miR-147a and LINC00847 wild type or mutant. **(O)** The levels of miR-147a was increased in Calu-3 and A549 cells after the transfection of si-LINC00847-1 and si-LINC00847-2. **(P)** Overexpression of miR-147a inhibited the expression of LINC00847. **p < 0.01.

Using the data of Lncatlas, it was discovered that LINC00847 was mainly expressed in the cytoplasm, suggesting this lncRNA may exhibit its tumor-promotive role via sponging miRNAs (Figures 5A,B) (19). Starbase 3.0 revealed that miR-147a contained a putative binding sites with LINC00847 (Figure 5C). Using subcellular fractionation, our group discovered that the nucleus and cytoplasm observed LINC00847 expressions (Figures 5D–F). The distinct down-regulation of miR-147a was also observed in NSCLC specimens (Figure 5G). NSCLC specimens with III-IV stages exhibited a lower level of miR-147a than those with I-II stages (Figure 5H). In four NSCLC cells, a significant decrease of miR-147a was observed compared with NHBE cells (Figure 5I). The diagnostic value of miR-147a was demonstrated by ROC assays (Figure 5J). Functional assays revealed that overexpression of miR-147a suppressed Calu-3 and A549 cell proliferation and invasion (Figures 5K,L). RNA-pull down demonstrated the association between LINC00847 and miR-147a (Figure 5M). More importantly, luciferase reporter assays provided evidence that miR-147a overexpression dramatically diminished the luciferase activity of LINC00847-Wt, but failed to change the relative luciferase activity possessed by LINC00847-Mut (Figure 5N). Besides, knockdown of LINC00847 resulted in the distinct promotion of miR-147a expression, while miR-147a overexpression suppressed the expression of LINC00847 (Figures 5O,P). Overall, our finding revealed that LINC00847 may exhibit its function in NSCLC progression via sponging miR-147a.

### IFITM1 Acted as a Target of miR-147a and LINC00847 Regulated IFITM1 Expression Through miR-147a in NSCLC Cells

**Figure 6:**
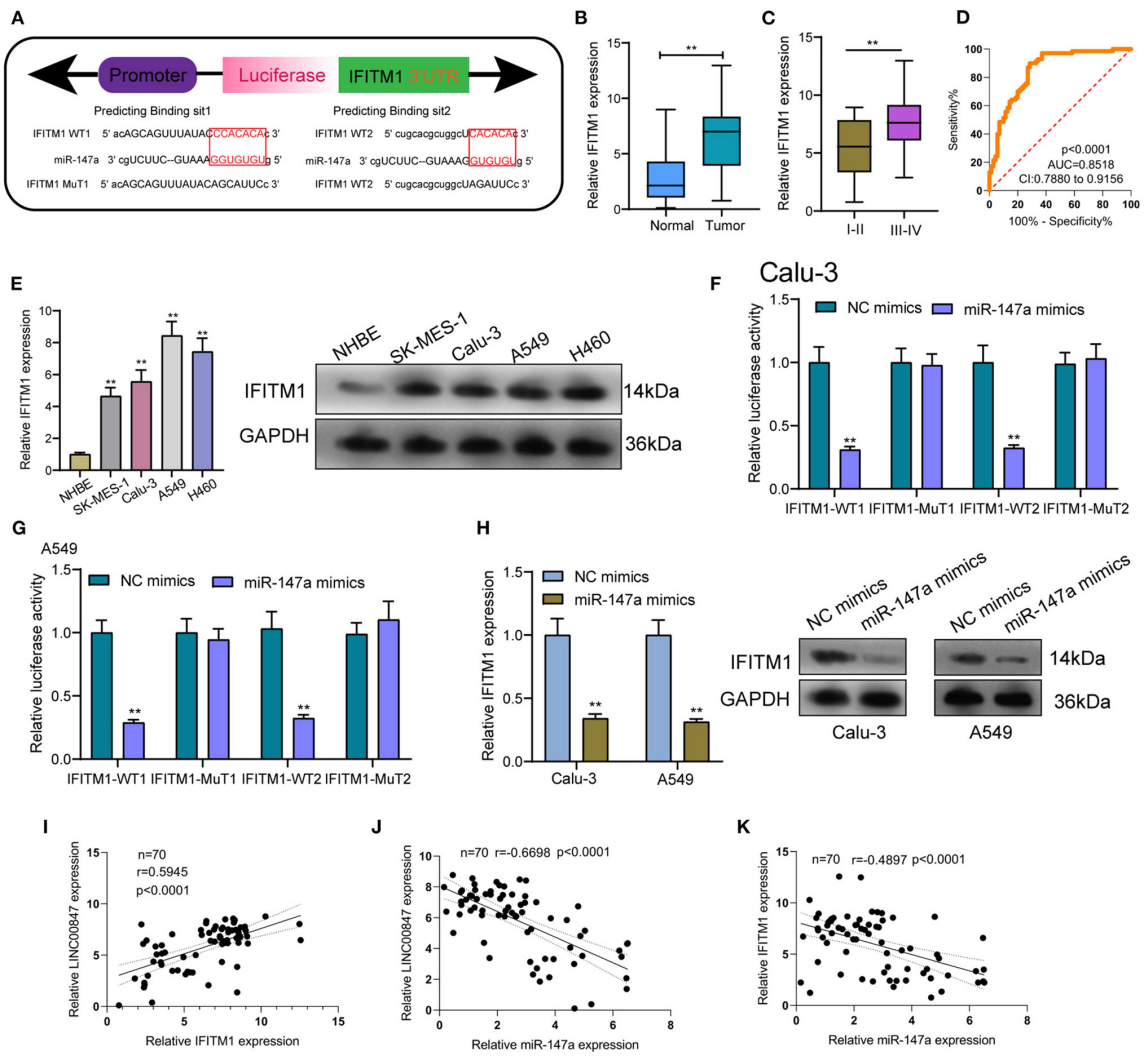
IFITM1 is a direct target of miR-147a. **(A)** Seed sequences of miR-147a against the wild-type or mutant 3'-UTR of IFITM1. **(B)** Expression levels of IFITM1 in clinical specimens of NSCLC. **(C)**The levels of IFITM1 in NSCLC specimens with different stages. **(D)** The area under the ROC curve was 0.8518. **(E)** the protein level of IFITM1 was increased in four NSCLC cell lines. **(F, G)** Relative luciferase activity was detected by luciferase assays in Calu-3 and A549 cells. **(H)** IFITM1 levels were increased after overexpression of miR-147a. **(I–K)** The correlation among LINC00847, miR-147a and IFITM1 expressions in our cohort was determined by Spearman's correlation analysis. **p < 0.01.

**Figure 7:**
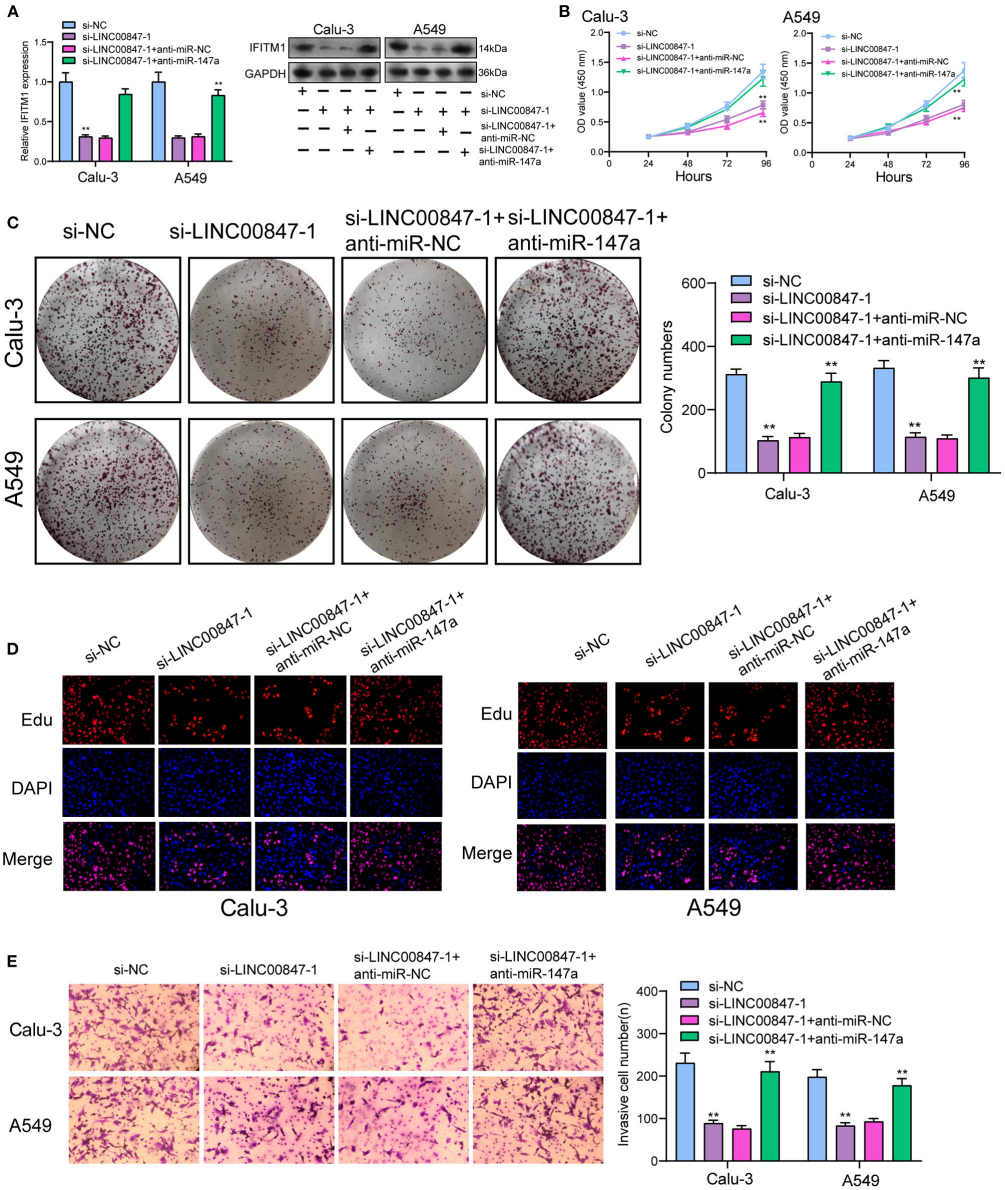
LINC00847 promoted the progression of NSCLC via sponging miR-146a to increase IFITM1. **(A)** The expression of IFITM1 at mRNA and protein level in Calu-3 and A549 cells after transfected with si-LINC00847-1, si-LINC00847-1/anti-miR-NC or si-LINC00847-1/anti-miR-147a. **(B)** CCK-8, **(C)** colon formation and **(D)** EdU were performed to detect cell proliferation. **(E)**The cell invasive ability of Calu-3 and A549 cells were assessed using Trans well-assays. **p < 0.01.

To explore the effects of miR-147a in the mechanisms and functions of LINC00847, we next sought to discover the downstream target of miR-147a using starbase algorithm, and found that IFITM1, a well-known oncogene in diverse cancer types, might be the possible target (Figure 6A). Then, the distinct overexpression of IFITM1 in NSCLC specimens and cells and its diagnostic value were also clinically demonstrated (Figures 6B–E). To confirm the binding between IFITM1 and miR-147a, luciferase activity assays were conducted. The results validated that co-transfection with wild-type1 (WT1) or wild-type2 (WT2) but not mutant-type1(MuT1) or mutant-type2 (MuT2) IFITM1 reporters led to significantly decreased luciferase activities in NSCLC cells, which demonstrated that miR-147a could directly target IFITM1 in NSCLC cells (Figures 6F,G). Furthermore, miR-147a overexpression suppressed the IFITM1 expressions at both mRNA and protein level (Figure 6H). Besides, the positive correlation between LINC00847 and IFITM1, and the negative correlation between LINC00847 and miR-147a and IFITM1 and LINC00847 were demonstrated in our cohort (Figures 6I–K). On the other hand, the results of rescue experiments revealed that LINC00847 knockdown resulted in the suppression of IFITM1 expression in Calu-3 and A549 cells, which was reversed because of the miR-147a knockdown (Figure 7A). According to a series of *in vitro* assays, in Calu-3 and A549 cells, miR-147a exhaustion weakened the proliferation and invasion suppression resulted from silencing LINC00847 (Figures 7B–E).

## Discussion

At present, the clinically common therapeutic method for lung cancer is chemotherapy, which, however, is only capable of prolonging the survival time of cancan patients but can not achieve the ultimate cure (20, 21). The identification of novel biomarkers for NSCLC patients is very important in the improvements of the clinical prognosis of patients. In this study, a novel NSCLC-related lncRNA, LINC00847 was identified, which showed overexpression in both NSCLC specimens and cell lines. Clinical studies also confirmed its diagnostic value in screening NSCLC specimens and provided evidence that high LINC00847 expression could lead to advanced clinical stages, distant metastasis and shorter OS and DFS. Our findings suggested LINC00847 being a new biomarker for the diagnosis and prognosis of NSCLC patients.

Although an increasing number of lncRNAs have been reported to exhibit a dysregulated level in different tumor specimens, the potential mechanisms remained largely unclear. Based on recent evidences, lncRNAs expression can be subjected to the transcription factors just like protein coding genes (22). In this study, we used JASPAR and PROMO to search the possible targets, finding that E2F1 may be a candidate with a high score. Then, we performed luciferase reporter assays, determining that E2F1 was capable of binding to the LINC00847 promoter region as well as inducing its transcription. The results of RT-PCR also confirmed the positive regulation between E2F1 and LINC00847. These essential data revealed that E2F1 activated LINC00847 translational expression to increase LINC00847 expression in NSCLC.

In recent years, growing studies have demonstrated the involvement of lncRNAs in the regulation of tumor growth and metastasis (23, 24). As LINC00847 was obviously overexpressed in NSCLC, we performed loss-of-function assays to explore its effects. As expected, we observed that LINC00847 knockdown weakened the proliferation, migration and invasion related to Calu-3 and A549 cells, suggesting it as a tumor pro motor in NSCLC. To explore the potential mechanisms by which LINC00847 promoted the progression of NSCLC, the localization in cancer cells was identified first because the function of LINC00847 was based on its subcellular localization. The results of biological information and subcellular fractionation confirmed the expression of LINC00847 in the cytoplasm as well as the nucleus, indicating cytosolic LINC00847 may act as a microRNA sponge. In addition, LINC00847 served as a ceRNA specific to miR-147a and sponged miR-147a. Previously, the tumor-suppressive roles of miR-147a in lung cancer and some other tumors have been demonstrated (25, 26). In functional exploration, we also observed that miR-147a presented a low expression in NSCLC and hindered NSCLC cell proliferation and invasion. As revealed, LINC00847 may serve as a tumor pro motor via sponging miR-147a.

Interferon-induced trans membrane protein 1 (IFITM1) acts as a member of the IFN-inducible trans membrane protein family and plays a functional role in the activity of cell adhesion signals and the anti-growth transduction (27). In recent years, growing studies have demonstrated that abnormal levels of IFITM1 affect the progression of several tumors types (28–30). In lung cancer, IFITM1 had the function of facilitating lung cancer cell proliferation and metastasis (31). In this study, bioinformatics analysis was used and identified IFITM1 as a potential target of miR-147a. Consistent with previous results, we also confirmed the distinct overexpression and the diagnostic value of IFITM1 in NSCLC. Further luciferase reporter assays demonstrated IFITM1 as a direct target of miR-147a. Besides, it was found that LINC00847 expression showed a positive relation to IFITM1 expression and a negative relation to miR-147a expression. In the rescue experiments, we observed that the down-regulation of miR-147a had the function of promoting NSCLC cells in terms of the proliferation and metastasis, that was hindered by LINC00847 knockdown. Overall, our findings suggested LINC00847 may exhibit its tumor-pro motive effects on the NSCLC progression by increasing IFITM1 expression via sponging miR-147a.

## Conclusions

LINC00847 expression was found to be increased in NSCLC and was activated by E2F1. LINC00847 may facilitate NSCLC cell proliferation and metastasis via sponging miR-147a and elevating IFITM1. The findings in the study help us to more deeply understand how lncRNAs affect NSCLC progression, how lncRNAs serve as an effective therapeutic target as well as how lncRNAs predict NSCLC prognosis.

## Data Availability Statement

The raw data supporting the conclusions of this article will be made available by the authors, without undue reservation.

## Ethics Statement

The studies involving human participants were reviewed and approved by Chongqing Public Health Medical Center. The patients/participants provided their written informed consent to participate in this study. The animal study was reviewed and approved by Chongqing Public Health Medical Center.

## Author Contributions

HL, L-xT, and MW wrote the main manuscript and analyzed the data. HL, Y-kC, QW, A-qS, and PH performed the experiments. HL, Y-kC, and L-xT designed the study. All authors read and approved the final manuscript. All authors contributed to the article and approved the submitted version.

## Conflict of Interest

The authors declare that the research was conducted in the absence of any commercial or financial relationships that could be construed as a potential conflict of interest.
